# Supplementary material for: The immune suppressive microenvironment affects efficacy of radio‐immunotherapy in brain metastasis
Source: EMBO Mol Med. 2021 Mar 23;13(5):e13412. doi: 10.15252/emmm.202013412 (PMC8103101; doi:10.15252/emmm.202013412)
Supplement: Supplementary file 1 — Appendix [file EMMM-13-e13412-s008.pdf]

Appendix

Appendix Fig S1 Intracranial and extracranial metastases in the 99LN-BrM model
Appendix Fig S2 Gating strategy for flow cytometry
Appendix Fig S3 T cell numbers in tumor-free and BrM-bearing mice
Appendix Fig S4 T cell numbers in peripheral blood following anti-CD4 and anti-CD8 antibody treatment

Appendix Table S1 Antibodies used for flow cytometry and sorting
Appendix Table S2 Antibodies used for histology
Appendix Table S3 Exact p-values for significant comparisons

Appendix Fig S1

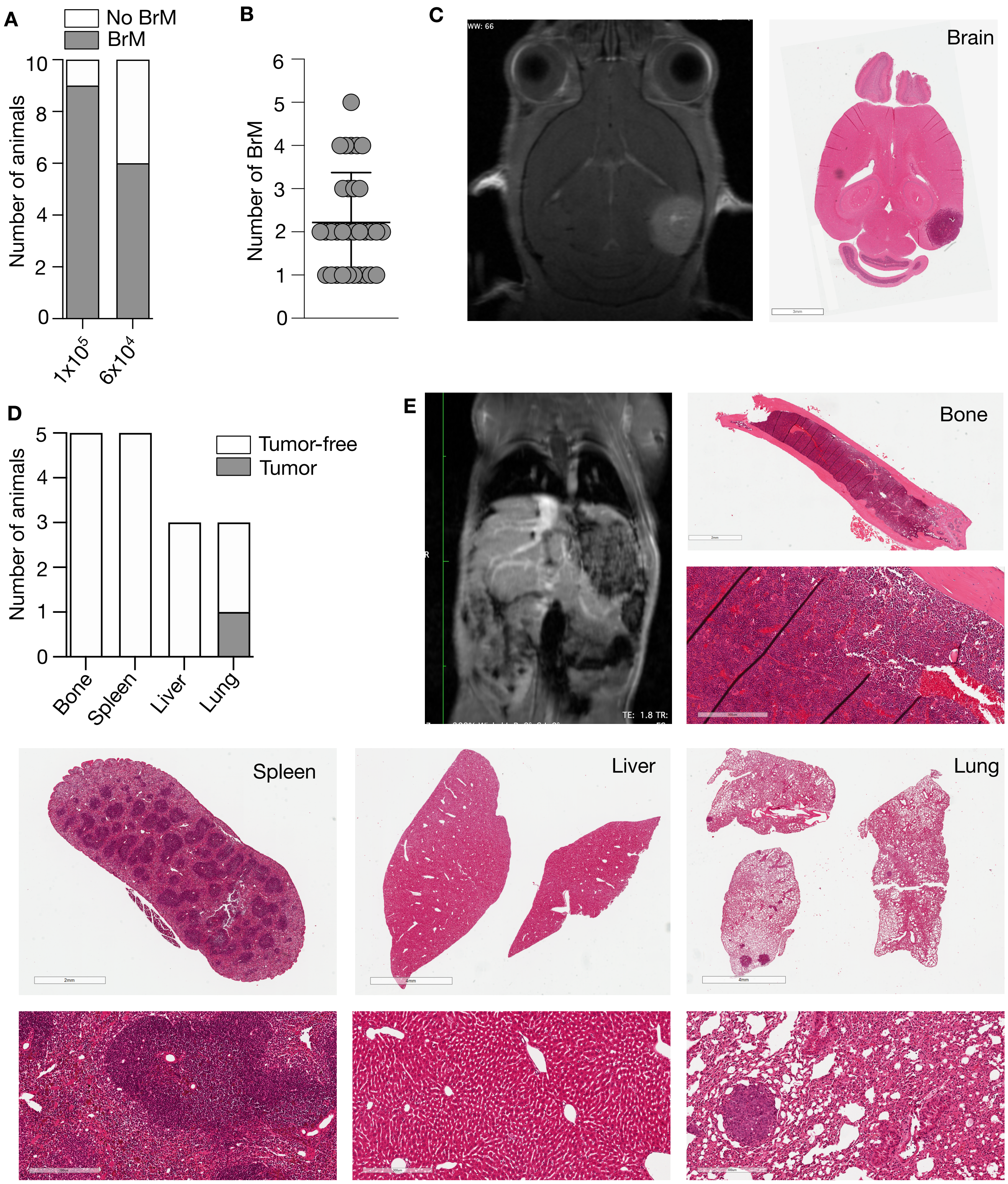

Appendix Fig S1. Intracranial and extracranial metastases in the 99LN-BrM model.

(**A**) Number of animals out of 10 animals which develop intracranial metastases after ICI of $1x10^5$ or $6x10^4$ 99LN-BrM cells.

(**B**) Number of BrM per animal after injection of $6x10^4$ 99LN-BrM cells (n=32).
(**C**) Representative images of MRI and HE-staining of 99LN-BrM.
(**D**) Number of animals with versus without extracranial metastases analyzed by HE staining (n=5 for bones and spleen, n=3 for liver and lung).
(**E**) Representative images of whole-body MRI and HE-sections of bones, spleen, liver and lung of the 99LN-BrM model.

Data information: Categorical data in (A+D) is represented as stacked columns. Numerical data in (B) is represented as mean ± SD.

Appendix Fig S2

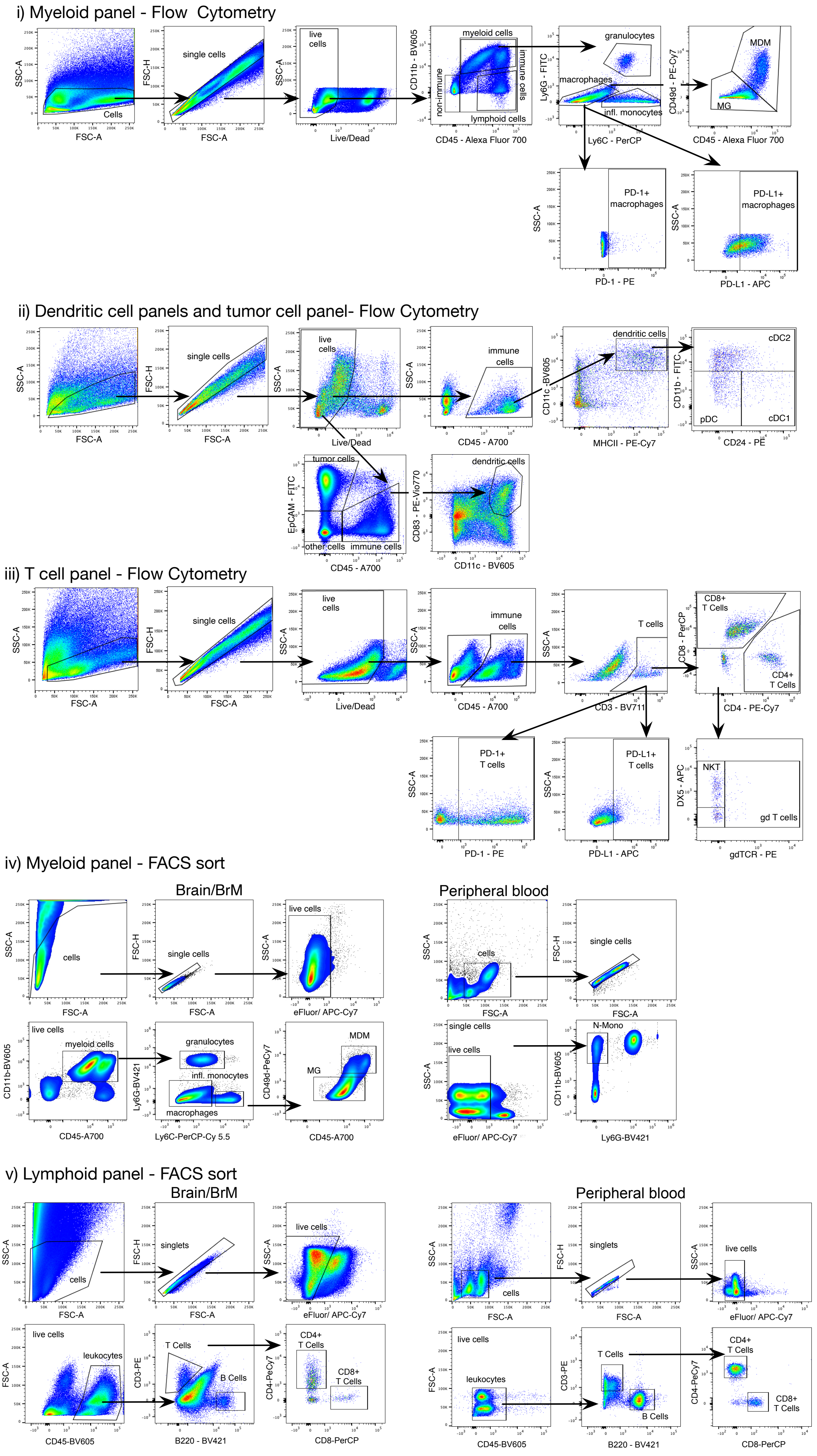

Appendix Fig S2. Gating strategy for flow cytometry. Representative FACS plots of the gating strategy for flow cytometry of CD45-EpCAM+ tumor cells and different myeloid and lymphoid immune cell populations from the 99LN-BrM model. The following marker combinations were used to discriminate immune cell populations: **i**) Microglia (MG; CD45+CD11b+Ly6ClowLy6G-CD49d-), monocyte-derived macrophages (MDM; CD45+CD11b+Ly6ClowLy6G-CD49d+), inflammatory monocytes (infl. monocytes; CD45+CD11b+Ly6ChighLy6G-) and granulocytes (CD45+CD11b+Ly6CmedLy6G+). **ii**) Tumor cells (EpCAM+CD45-), dendritic cells (DC; CD45+CD11c+CD83+ or CD45+CD11c+MHCII+), conventional DC type 1 (cDC1; CD45+CD11c+MHCII+CD11b-CD24+), conventional DC type 2 (cDC2; CD45+CD11c+MHCII+CD11b+), and other/plasmacytoid DC (pDC; CD45+CD11c+MHCII+CD11b-CD24-). **iii**) CD4+ T cells (CD45+CD3+CD4+), CD8+ T cells (CD45+CD3+CD8+), γδ-T cells (CD45+CD3+CD4-CD8-γδTCR+), and CD4-CD8- NK-T cells (CD45+CD3+CD4-CD8-DX5+). Depending on the experiment requirements this strategy was combined with analysis of PD-1/PD-L1 expression. For the sorting of cell populations for RNA-sequencing, the following marker combinations were applied: **iv**) myeloid cell populations of brain/BrM the same as in **i**) and blood monocytes (N-Mono; CD11b+Ly6G-). **v**) Lymphoid cells in Brain/BrM and peripheral blood: B cells (CD45+B220+CD3-), CD4+ T cells (CD45+B220-CD3+CD4+), and CD8+ T cells (CD45+B220-CD3+CD8+).

Appendix Fig 3

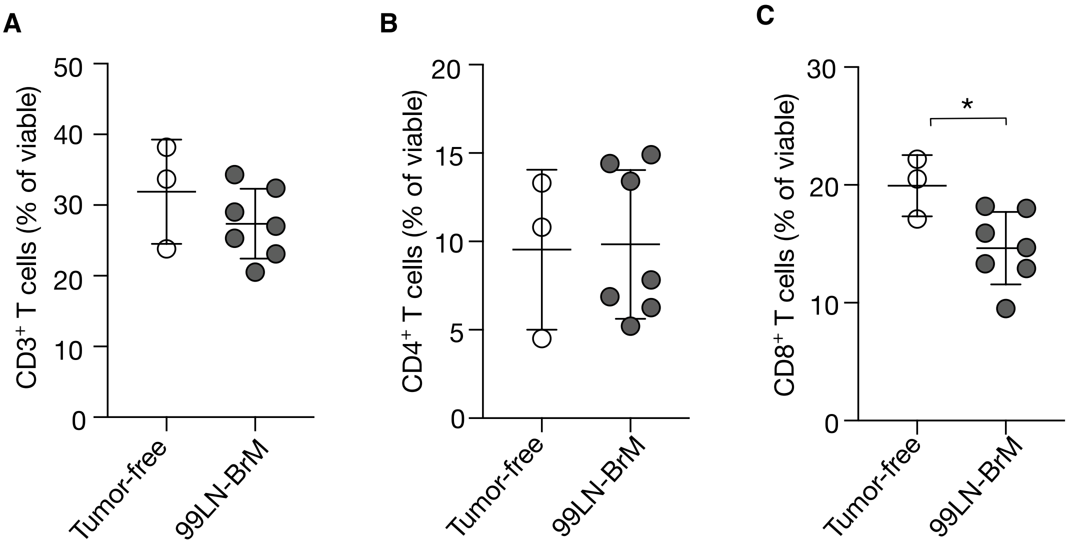

Appendix Fig S3. T cell numbers in tumor-free and BrM-bearing mice.
(A-C) Flow cytometric analysis of different T cell populations in peripheral blood from tumor-free and 99LN-BrM mice showing CD3+ T cells (A), CD4+ T cells (B) and CD8+ T cells (C) with n= 3 for tumor-free and n=7 for 99LN-BrM.
Data information: Data is represented as scatter dot plot with lines mean ± SD. P-values were obtained by unpaired t test with $^{*}P<0.05$.

Appendix Fig S4

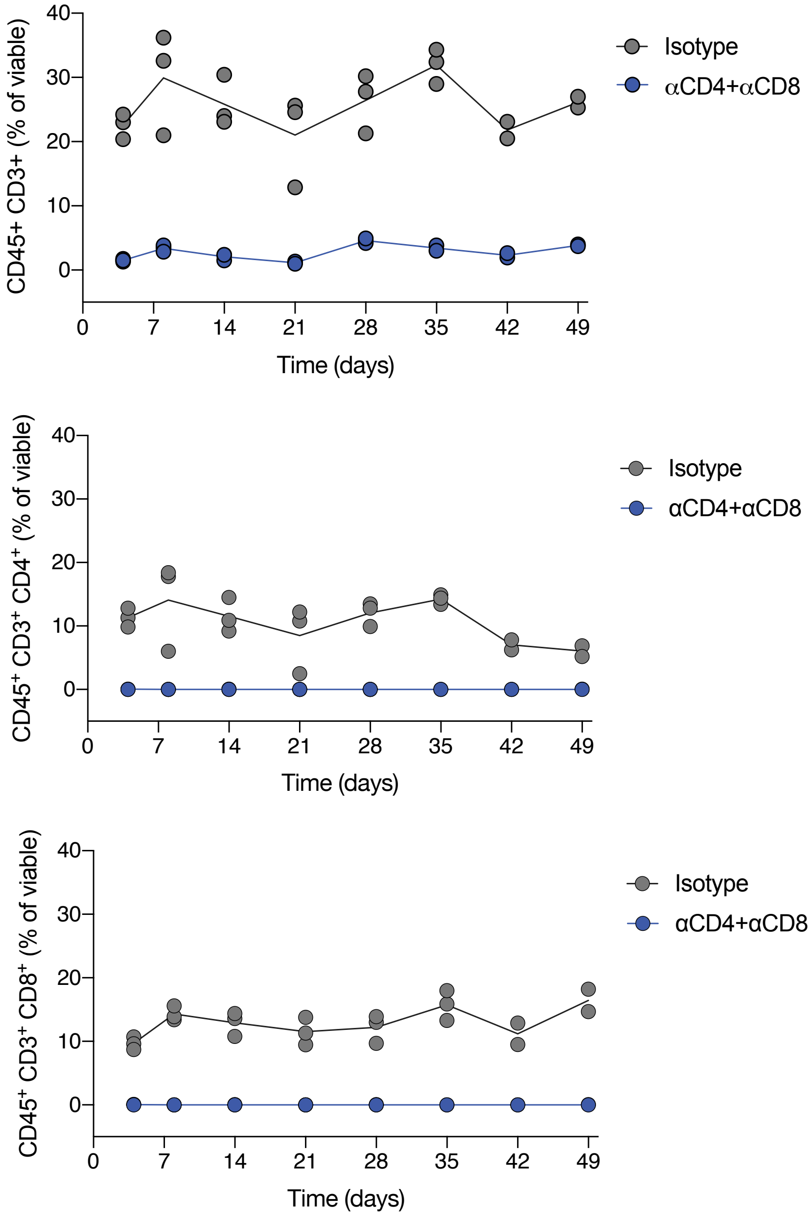

Appendix Fig S4. T cell numbers in peripheral blood following anti-CD4 and anti-CD8 antibody treatment.

(**A-C**) Monitoring of total T cells (A), CD4+ T cells (B) and CD8+ T cells (C) in the blood of mice treated with isotype or CD4/CD8 depleting antibodies via flow cytometry. (n=2-3 for isotype and depletion group).

Appendix Table S1 Markers and antibodies used for flow cytometry

**Flow cytometry: PD-1 / PD-L1 overview panel**						
Antigen /marker	*Fluorochrome*	*Species/ host*	*Clone*	*Dilution*	*Vendor*	*Catalog no.*
eFluor	eFluor 780			1:100	eBioscience	65086514
CD3	PE	Human	REA641	1:500	Miltenyi Biotec	130109879
CD45	PerCP	Rat	30-F11	1:200	eBioscience	45045-80
CD11b	BV605	Rat	M1/70	1:1,000	BD Biosciences	563015
CD326	FITC	Rat	G8.8	1:500	eBioscience	11579180
PD-1	BV421	Hamster	J43	1:500	BD Biosciences	562584
PD-L1	APC	Rat	MIH5	1:200	BD Biosciences	564715
**Flow cytometry: Myeloid panel**						
Antigen /marker	*Fluorochrome*	*Species*	*Clone*	*Dilution*	*Vendor*	*Catalog no.*
LIVE/DEAD	Fix Blue	**-**	**-**	1:500	Thermo Fisher	L34962
CD45	Alexa Fluor 700	Rat	30-F11	1:500	BioLegend	103128
CD11b	BV605	Rat	M1/70	1:1,000	BD Biosciences	563015
Ly6C	PerCP-Cy5.5	Rat	HK1.4	1:250	BioLegend	128011
Ly6G	FITC	Rat	1A8	1:500	BioLegend	127605
CD49d	PE-Cy7	Rat	R1-2	1:400	BioLegend	103618
PD-1	PE	Rat	29F.1A12	1:100	BioLegend	135205
PD-L1	APC	Rat	MIH5	1:100	BD Biosciences	564715
**Flow cytometry: T cell panel 1**						
Antigen /marker	*Fluorochrome*	*Species*	*Clone*	*Dilution*	*Vendor*	*Catalog no.*
LIVE/DEAD	Fix Blue	-	-	1:500	Thermo Fisher	L34962
CD45	AlexaFluor700	Rat	30-F11	1:500	BioLegend	103128
CD3ε	BV711	Hamster	1452C11	1:500	BD Biosciences	563123
CD4	PE-Vio770	Rat	GK1.5	1:100	Miltenyi Biotec	130102784
CD8α	PerCP-Cy5.5	Rat	53-6.7	1:100	BD Biosciences	561109
γδ-TCR	PE	Hamster	GL3	1:200	BioLegend	118108
DX5	APC	Rat	DX5	1:100	BioLegend	108910
**Flow cytometry: T cell panel 2**						
Antigen /marker	*Fluorochrome*	*Species*	*Clone*	*Dilution*	*Vendor*	*Catalog no.*
LIVE/DEAD	Fix Blue	-	-	1:500	Thermo Fisher	L34962
CD45	FITC	Rat	30-F11	1:500	BD Biosciences	553080
CD3ε	BV711	Hamster	1452C11	1:500	BD Biosciences	563123
CD4	PE-Vio770	Rat	GK1.5	1:500	Miltenyi Biotec	130102784
CD8α	PerCP-Cy5.5	Rat	53-6.7	1:250	BD Biosciences	561109
PD-1	PE	Rat	29F.1A12	1:100	BioLegend	135205
PD-L1	APC	Rat	MIH5	1:100	BD Biosciences	564715
**Flow cytometry: Dendritic cell / tumor cell panel**						
Antigen/ marker	*Fluorochrome*	*Species*	*Clone*	*Dilution*	*Vendor*	*Catalog no.*
LIVE/DEAD	Fix Blue	-	-	1:500	Thermo Fisher	L34962
CD45	AlexaFluor700	Rat	30-F11	1:500	BioLegend	103128
CD326	FITC	Rat	G8.8	1:1,000	eBioscience	11579180
CD83	PE-Vio770	Human	REA304	1:500	Miltenyi Biotec	130104476
CD11c	BV605	Hamster	N418	1:250	BioLegend	117333
PD-1	PE	Rat	29F.1A12	1:100	BioLegend	135205
PD-L1	APC	Rat	MIH5	1:100	BD Biosciences	564715
**Flow cytometry: cDC1 / cDC2 panel**						
Antigen/ marker	*Fluorochrome*	*Species*	*Clone*	*Dilution*	*Vendor*	*Catalog no.*
LIVE/DEAD	Fix Blue	-	-	1:500	Thermo Fisher	L34962
CD45	AlexaFluor700	Rat	30-F11	1:500	BioLegend	103128

CD11b	FITC	Rat	M1/70	1:1,000	BD Biosciences	553310
CD11c	BV605	Hamster	N418	1:250	BioLegend	117333
MHCII	PE-Cy7	Rat	M5/114.1 5.2	1:500	BioLegend	107629
CD24	PE	Rat	30-F1	1:250	BioLegend	138503
**Flow cytometry: T cell activation assay I**						
Antigen/ marker	*Fluorochrome*	*Species*	*Clone*	*Dilution*	*Vendor*	*Catalog no.*
LIVE/DEAD	Fix Blue	-	-	1:500	Thermo Fisher	L34962
CD45	AlexaFluor700	Rat	30-F11	1:500	BioLegend	103128
CD11b	FITC	Rat	M1/70	1:1,000	BD Biosciences	553310
CD69	BV605	Hamster	H1.2F3	1:500	BioLegend	104529
**Flow cytometry: T cell activation assay II**						
Antigen/ marker	*Fluorochrome*	*Species*	*Clone*	*Dilution*	*Vendor*	*Catalog no.*
LIVE/DEAD	Fix Blue	-	-	1:500	Thermo Fisher	L34962
CD45	AlexaFluor700	Rat	30-F11	1:500	BioLegend	103128
CD4	PE-Vio770	Rat	GK1.5	1:200	Miltenyi Biotec	130102784
CD8α	PerCP-Cy5.5	Rat	53-6.7	1:250	BD Biosciences	561109
Grzmb	PE-eFluor 610	Rat	NGZB	1:200	Thermo Fisher	61-8898-82
IFNg	FITC	Rat	XMG1.2	1:200	BD Biosciences	554411
**Flow sort: Myeloid panel**						
LIVE/DEAD	eFluor-780	-	-	1:50	Thermo Fisher	65-0865-14
CD45	AlexaFluor700	Rat	30-F11	1:500	BioLegend	103128
CD11b	BV605	Rat	M1-70	1:1000	BD Biosciences	563015
Ly6C	PerCP-Cy5.5	Rat	HK1.4	1:250	BioLegend	128012
Ly6G	BV421	Rat	1A8	1:500	BD Biosciences	562737
CD49d	Pe-Cy7	Rat	R1-2	1:500	BioLegend	103618
**Flow sort: Lymphoid panel**						
LIVE/DEAD	eFluor-780	-	-	1:50	Thermo Fisher	65-0865-14
CD45	BV605	Rat	30-F11	1:500	BD Biosciences	563053
CD3	PE	Cell line	REA641	1:500	Miltenyi	130-120-826
B220	BV421	Rat	RA3-6B2	1:300	BD Biosciences	562922
CD4	PE-Vio770	Rat	GK1.5	1:500	Miltenyi	130-124-712
CD8	PerCP-Cy5.5	Rat	53-6.7	1:500	BD Biosciences	561109

Appendix Table S2 Antibodies used for histology

Antigen	*Species*	*Clonality-Clone*	*Dilution*	*Vendor*	*Catalog no.*
CD3	Rabbit	EPR20752	1:2,800	Abcam	ab215212
CD8	Rabbit	D4W2Z	1:500	Cell Signaling	98941S
DCIR2	Rat	33D1	1:500	eBioscience	14588482
Epcam	Rabbit	polyclonal	1:500	Abcam	ab71916
Iba1	Rabbit	polyclonal	1:1,000	Novus Biologicals	NBP2-19019
FoxP3	Rabbit	polyclonal	1:500	Abcam	ab54501
PD-1	Goat	polyclonal	1:100	R&D Systems	AF1021
PD-L1	Rabbit	E1L3N	1:200	Cell Signaling	13684S
TMEM119	Guinea pig	polyclonal	1:1,000	SYSY	400004
**Phenoptics Multiplexed Immunofluorescence Panel**					
Antigen	*Species*	*Opal*	*Dilution*	*Vendor*	*Catalog no.*
CD4	Rabbit	570	1:450	abcam	Ab183685
CD8a	Rabbit	520	1:450	Cell Signalling	989415
EpCAM	Rabbit	620	1:180	Abcam	ab71916
CD206	Rabbit	690	1:450	Abcam	ab64693
TMEM119	Rabbit	480	1:100	Abcam	ab209064
Iba1	Rabbit	780	1:200	Wako Chemicals	019-19741

Appendix Table S3 Exact p-values for significant comparisons

Figure panel	Comparison	Statistical test	p-value
2D	Isotype vs. aCD4+aCD8	Unpaired t test	*P*=0.0393
2H	Padj. for the indicated genes can be found in the Data source file for Fig 2		
3A	Tumor cells vs. T cells	Unpaired t test	P=0.0074
	Myeloid cells vs. T cells	Unpaired t test	P=0.0089
3C	Tumor-free vs. BrM	Unpaired t test	*P*=0.0487
3D	MG vs. Granu	Unpaired t test	*P*=0.0056
	MG vs. Monocytes	Unpaired t test	*P*<0.0001
	MG vs. MDM	Unpaired t test	*P*<0.0001
4N	Ctrl vs. WBRT	Unpaired t test	*P*=0.0261
4Q	Ctrl vs. WBRT	Unpaired t test	*P*=0.0111
4R	Ctrl vs. WBRT	Unpaired t test	*P*=0.0394
5C	Ctrl BrM vs. Ctrl CLN	Non-parametric test (Mann Whitney test)	*P*=0.0079
	WBRT BrM vs. Ctrl CLN	Non-parametric test (Mann Whitney test)	*P*=0.0159
5D	Ctrl BrM vs. Ctrl CLN	Non-parametric test (Mann Whitney test)	*P*=0.0079
	WBRT BrM vs. Ctrl CLN	Non-parametric test (Mann Whitney test)	*P*=0.0159
5G	Ctrl BrM vs. Ctrl CLN	Unpaired t test	*P*=0.0021
	WBRT BrM vs. Ctrl CLN	Unpaired t test	*P*=0.0002
6D	Isotype vs. WBRT+aPD1	Unpaired t test	*P*=0.0014
	WBRT vs. WBRT+aPD1	Unpaired t test	*P*=0.0281
6G	Isotype vs. WBRT+aPD1	Log-Rank test	*P*=0.0473
	WBRT vs. WBRT+aPD1	Log-Rank test	*P*=0.0494
7C	Isotype vs. aPD1	Non-parametric test (Mann Whitney test)	*P*=0.0288
7D	Multiple comparisons	Two-way ANOVA; Row factor	*P*=0.0468
	Multiple comparisons	Two-way ANOVA; Colums factor	*P*=0.0459
7F	Isotype vs. aPD1	Unpaired t test	*P*=0.0478
7G	Multiple comparisons	Two-way ANOVA; Row factor	n.s.
	Multiple comparisons	Two-way ANOVA; Colums factor	P<0.0001
7H	Multiple comparisons	Two-way ANOVA; Row factor	*P*=0.0168
	Multiple comparisons	Two-way ANOVA; Colums factor	*P*=0.0011
7J	Baseline vs. BMDM cond	Paired t test	*P*=0.0310
	Baseline vs. BMDM+99LN	Paired t test	*P*=0.0276
	Baseline vs. EOC2+99LN	Paired t test	*P*=0.0341
7K upper left	Baseline vs. BMDM+aPD1	Paired t test	*P*=0.0021
	Baseline vs. cond BMDM	Paired t test	*P*=0.0313
	Baseline vs. EOC2+99LN	Paired t test	*P*=0.0062
	Baseline vs. EOC2+99LN+aPD1	Paired t test	*P*=0.0394
	Baseline vs. BMDM+99LN	Paired t test	*P*=0.0023
	Baseline vs. BMDM+99LN+aPD1	Paired t test	*P*=0.0190
7K upper right	Baseline vs. EOC2+99LN	Paired t test	*P*=0.0271
	Baseline vs. BMDM+99LN	Paired t test	*P*=0.0111
7K lower left	BMDM+99LN-aPD1 vs. BMDM+99LN+aPD1	Paired t test	*P*=0.0371
EV4B day 14	Isotype vs. WBRT	Unpaired t test	P=0.0020
	WBRT vs. aPD1	Unpaired t test	P=0.0114
	aPD1 vs. WBRT+aPD1	Unpaired t test	P=0.0038
	Isotype vs. WBRT+aPD1	Unpaired t test	P=0.0003
EV4B day 28	aPD1 vs. WBRT+aPD1	Unpaired t test	P=0.0362
	Isotype vs. WBRT+aPD1	Unpaired t test	P=0.0272
EV4C	aPD1 CD3 high vs CD3 low	Log-Rank test	P=0.0039
