# Supplementary material for: The immune suppressive microenvironment affects efficacy of radio‐immunotherapy in brain metastasis
Source: EMBO Mol Med. 2021 Mar 23;13(5):e13412. doi: 10.15252/emmm.202013412 (PMC8103101; doi:10.15252/emmm.202013412)
Supplement: Supplementary file 2 — Expanded View Figures PDF [file EMMM-13-e13412-s010.pdf]

Expanded View Figures

Figure EV1. Transcription programs in tumor-associated myeloid and lymphoid populations.

A Expression of MG and monocyte/MDM restricted genes in tumor-associated myeloid cells and control cells from tumor-free animals ($n = 3$; note: individual samples showed no expression for specific genes and are indicated on the *x*-axis). Values are depicted as normalized counts.

B Expression of T- and B-cell restricted genes in tumor-infiltrating lymphocytes and blood lymphocytes from tumor-free animals ($n = 3$; note: individual samples showed no expression for specific genes and are indicated on the *x*-axis). Values are depicted as normalized counts.

C Amount of significant DEG (cutoff: base mean (BM) > 20, *P*adj < 0.05) in different cell types based on RNAseq data of tumor-associated vs. control cell types ($n = 3$).

D Euler plots depict shared and unique DEGs (cutoff: BM > 20, *P*adj. < 0.05) in each cell type based on RNAseq data from tumor-associated vs. control cell types ($n = 3$).

E Unsupervised clustering of the top 100 DEG in control MG vs. TAM-MG, BL-Mono vs. TAM-MDM. BL-CD4 vs. TIL-CD4 and BL-CD8 vs. TIL-CD8 (cutoff: BM > 20, *P*adj. < 0.05) ($n = 3$). Selected genes are annotated.

Data information: Adjusted *P*-values (*P*adj.) in (C–E) were obtained by Wald test and corrected for multiple testing using the Benjamini and Hochberg method.
Source data are available online for this figure.

 © 2021 The Authors

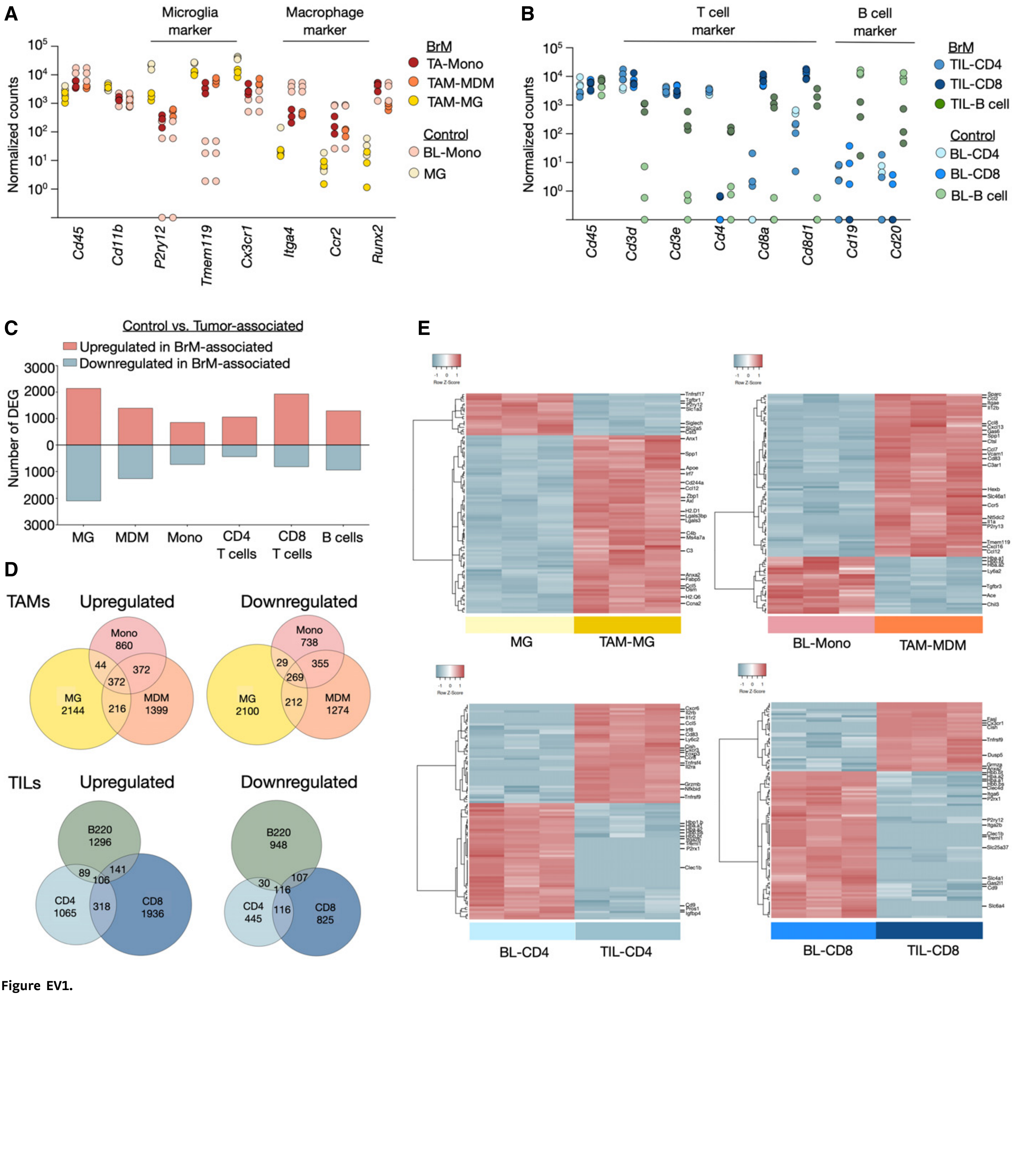
A
Microglia marker
Macrophage marker
Normalized counts
Cd45
Cd11b
P2ry12
Tmem119
Cx3cr1
Itga4
Ccr2
Runx2
BrM
TA-Mono
TAM-MDM
TAM-MG
Control
BL-Mono
MG
B
T cell marker
B cell marker
Cd3d
Cd3e
Cd4
Cd8a
Cd8d1
Cd19
Cd20
TIL-CD4
TIL-CD8
TIL-B cell
BL-CD4
BL-CD8
BL-B cell
C
Control vs. Tumor-associated
Upregulated in BrM-associated
Downregulated in BrM-associated
Number of DEG
MG
MDM
Mono
CD4 T cells
CD8 T cells
B cells
D
TAMs
Upregulated
Downregulated
Mono 860
MG 2144
MDM 1399
Mono 738
MG 2100
MDM 1274
TILs
B220 1296
CD4 1065
CD8 1936
B220 948
CD4 445
CD8 825
E
MG
TAM-MG
BL-Mono
TAM-MDM
BL-CD4
TIL-CD4
BL-CD8
TIL-CD8

Figure EV1.

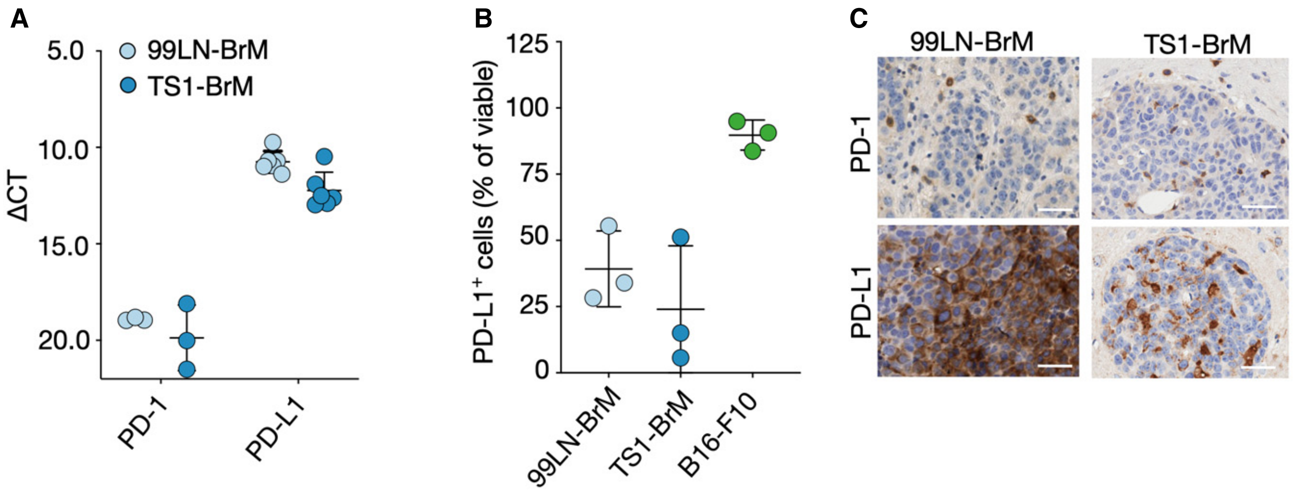

Figure EV2. Expression of PD-1 and PD-L1 by breast cancer BrM.

A Expression of PD-1 and PD-L1 in the BrM cell lines 99LN-BrM and TS1-BrM measured by qRT-PCR (n = 6 for PD-L1 and n = 3 for PD-1).

B Proportion of 99LN-BrM, TS1-BrM and B16-F10 cells positive for PD-L1 measured by flow cytometry (n = 3).

C Representative IHC images of PD-1 and PD-L1 expressing cells in 99LN-BrM and TS1-BrM (Scale bars = 50 μm).

Data information: Data in (A + B) are presented as scattered dot plot with lines at mean ± SD.
Source data are available online for this figure.

Figure EV3. Top T-cell clones and clonal overlap in BrM and CLN.

A Bar graphs depict the percentage of the top clone from the total T-cell pool in the individual samples.

B Productive clonality of BrM samples plotted against total templates to exclude a correlation.

C Comparison of relative abundance of clones with a specific frequency in control and WBRT BrM samples. Values are depicted as scatter plot in bars indicating mean with 95% confidence interval (CI).

D Venn diagrams depict the overlap of the top 100 clones in BrM and CLN samples from the individual mice.

E Overlap of the top 100 clones of BrM samples in the control and WBRT group.

Data information: n = 5 for BrM and CLN from untreated mice, n = 4 for BrM, and n = 5 for CLN from irradiated mice.
Source data are available online for this figure.

© 2021 The Authors

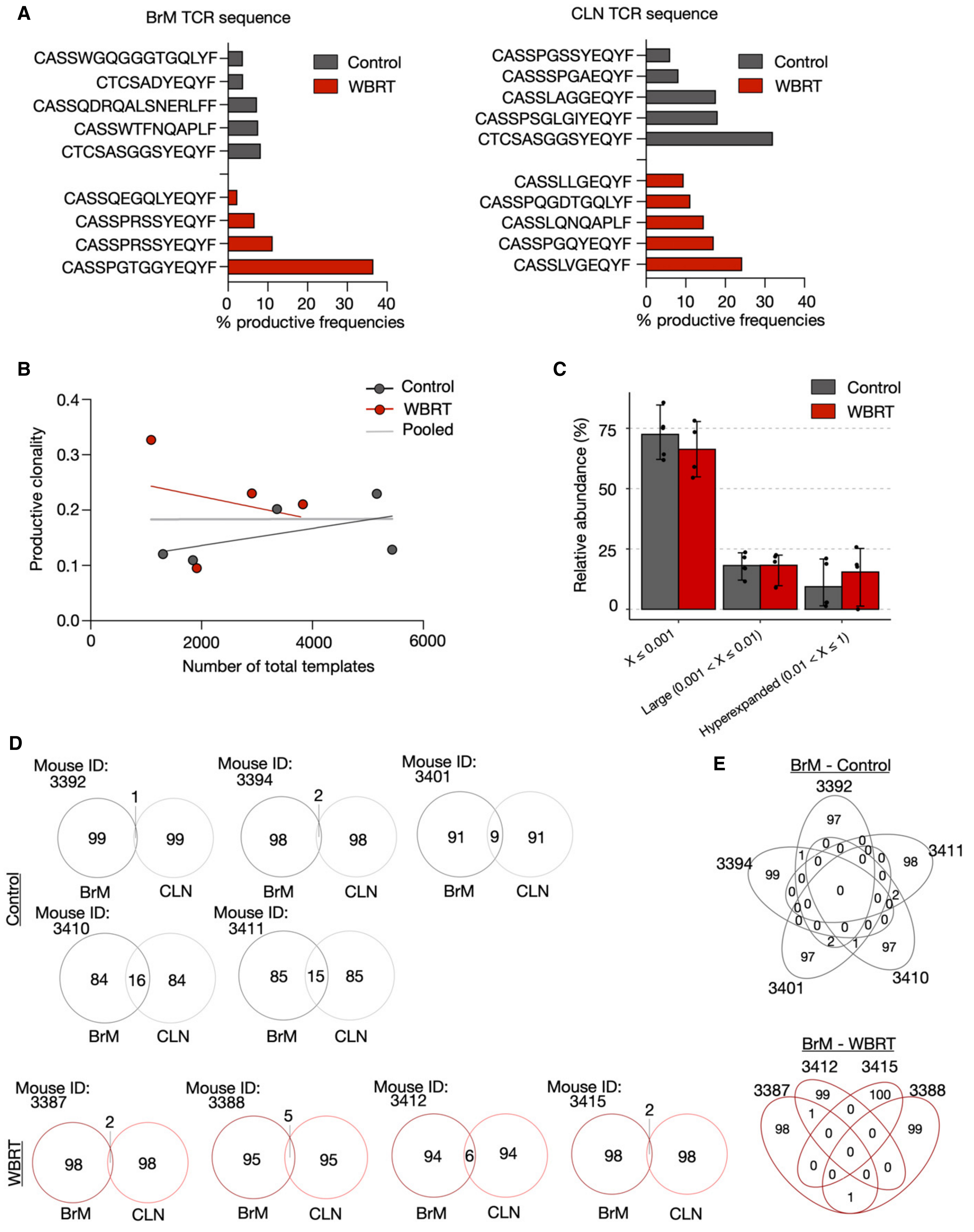
A
BrM TCR sequence
CASSWGQGGGTGQLYF
CTCSADYEQYF
CASSQDRQALSNERLFF
CASSWTFNQAPLF
CTCSASGGSYEQYF
CASSQEGQLYEQYF
CASSPRSSYEQYF
CASSPRSSYEQYF
CASSPGTGGYEQYF
Control
WBRT
0 10 20 30 40
% productive frequencies
CLN TCR sequence
CASSPGSSYEQYF
CASSSPGAEQYF
CASSLAGGEQYF
CASSPSGLGIYEQYF
CTCSASGGSYEQYF
CASSLLGEQYF
CASSPQGDTGQLYF
CASSLQNQAPLF
CASSPGQYEQYF
CASSLVGEQYF
Control
WBRT
0 10 20 30 40
% productive frequencies
B
Control
WBRT
Pooled
Productive clonality
0.0 0.1 0.2 0.3 0.4
0 2000 4000 6000
Number of total templates
C
Control
WBRT
Relative abundance (%)
0 25 50 75
X ≤ 0.001
Large (0.001 < X ≤ 0.01)
Hyperexpanded (0.01 < X ≤ 1)
D
Control
Mouse ID: 3392
99 1 99
BrM CLN
Mouse ID: 3394
98 2 98
BrM CLN
Mouse ID: 3401
91 9 91
BrM CLN
Mouse ID: 3410
84 16 84
BrM CLN
Mouse ID: 3411
85 15 85
BrM CLN
WBRT
Mouse ID: 3387
98 2 98
BrM CLN
Mouse ID: 3388
95 5 95
BrM CLN
Mouse ID: 3412
94 6 94
BrM CLN
Mouse ID: 3415
98 2 98
BrM CLN
E
BrM - Control
3392
3411
3394
3401
3410
BrM - WBRT
3412 3415
3387
3388

Figure EV3.

Figure EV4. Efficacy of radio-immunotherapy in BrM.

A Number and volume of BrM in each treatment group on d-1.
B Waterfall plots depict percent increase in tumor volume of individual mice in each treatment group on days 14 and 28.
C Kaplan–Meier curves show symptom-free survival in the respective treatment groups when mice were categorized as CD3 T-cell high or low subgroups.

Data information: ($n = 8$, $n = 7$, $n = 8$, $n = 9$ for isotype, WBRT, αPD-1, WBRT + αPD-1 group, respectively). Data in (A) are represented as scatter dot plot with line at mean ± SD. *P*-values were obtained by unpaired *t*-test in (B) and log-rank test in (C) with $^*P < 0.05$, $^{**}P < 0.01$, and $^{***}P < 0.001$. Exact *P*-values can be found in Appendix Table S3.
Source data are available online for this figure.

 © 2021 The Authors

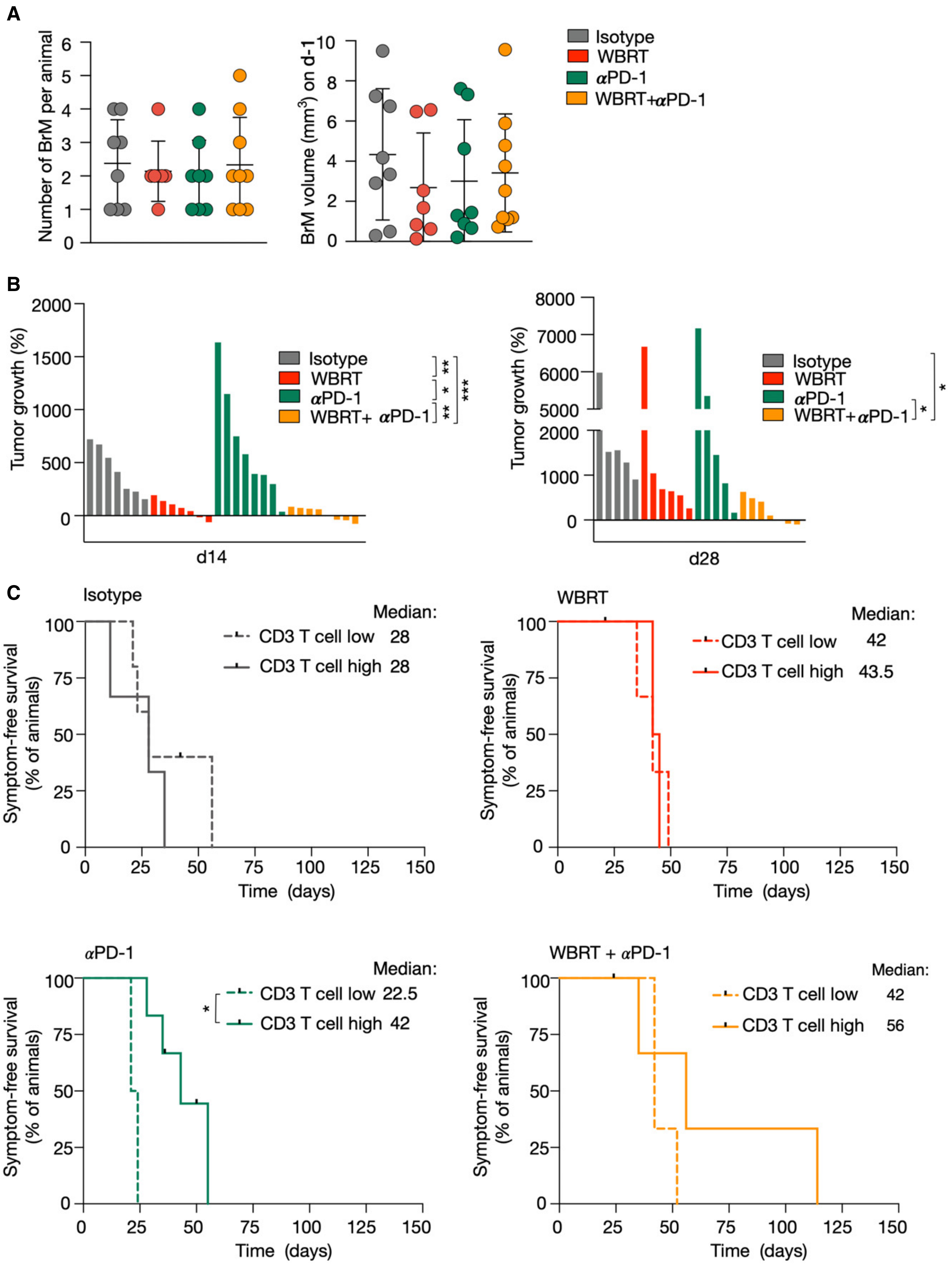
A
Number of BrM per animal
BrM volume (mm3) on d-1
Isotype
WBRT
αPD-1
WBRT+αPD-1
B
Tumor growth (%)
Isotype
WBRT
αPD-1
WBRT+ αPD-1
d14
d28
C
Isotype
Median:
CD3 T cell low 28
CD3 T cell high 28
Symptom-free survival (% of animals)
Time (days)
WBRT
Median:
CD3 T cell low 42
CD3 T cell high 43.5
αPD-1
Median:
CD3 T cell low 22.5
CD3 T cell high 42
WBRT + αPD-1
Median:
CD3 T cell low 42
CD3 T cell high 56

Figure EV4.

Figure EV5. Spatial distribution of immune cells in BrM after treatment.

A Representative halo spatial plot showing the spatial distribution of cell types with indicated marker combination in 99LN-BrM in response to treatment with WBRT, αPD-1, and WBRT + αPD-1.

B HALO proximity histograms depict the distance in μm between the indicated cell types in BrM lesions for individual mice in response to treatment (*n* = 3 per condition).

Source data are available online for this figure.

 © 2021 The Authors

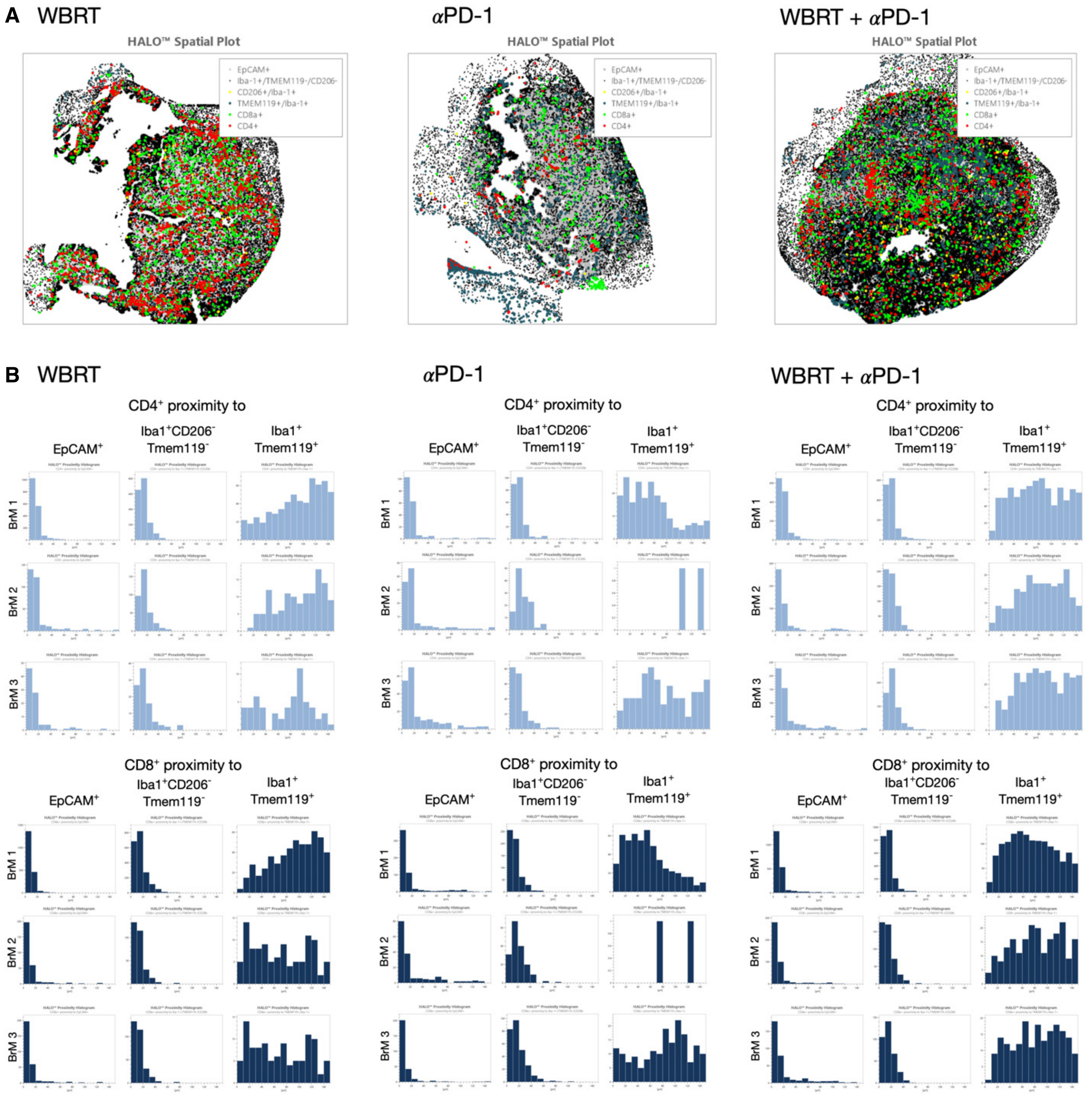
A WBRT
αPD-1
WBRT + αPD-1
HALO™ Spatial Plot
EpCAM+
Iba-1+/TMEM119-/CD206-
CD206+/Iba-1+
TMEM119+/Iba-1+
CD8a+
CD4+
B WBRT
αPD-1
WBRT + αPD-1
CD4+ proximity to
EpCAM+
Iba1+CD206- Tmem119-
Iba1+ Tmem119+
BrM 1
BrM 2
BrM 3
CD8+ proximity to
HALO™ Proximity Histogram

Figure EV5.
