# Supplementary figures and images for: The immune suppressive microenvironment affects efficacy of radio‐immunotherapy in brain metastasis
Source: EMBO Mol Med. 2021 Mar 23;13(5):e13412. doi: 10.15252/emmm.202013412 (PMC8103101; doi:10.15252/emmm.202013412)

Figure 2C - uncropped images

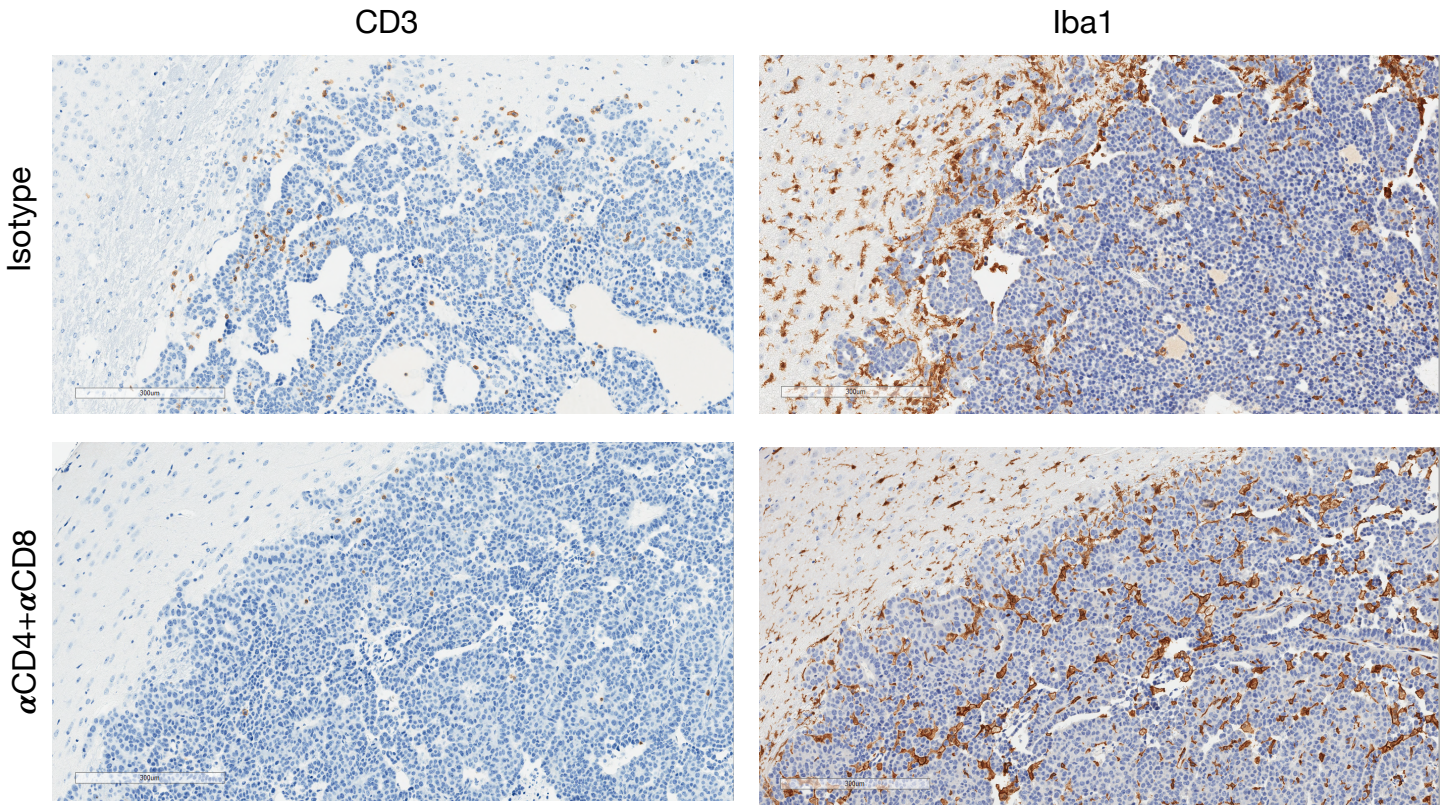

Supplement: Supplementary file 8 — Source Data for Figure 2 [file EMMM-13-e13412-s012.zip › EMM-2020-13412-V2-Figure_2_Source_Data-sd.pdf]

Figure 4 - uncropped images

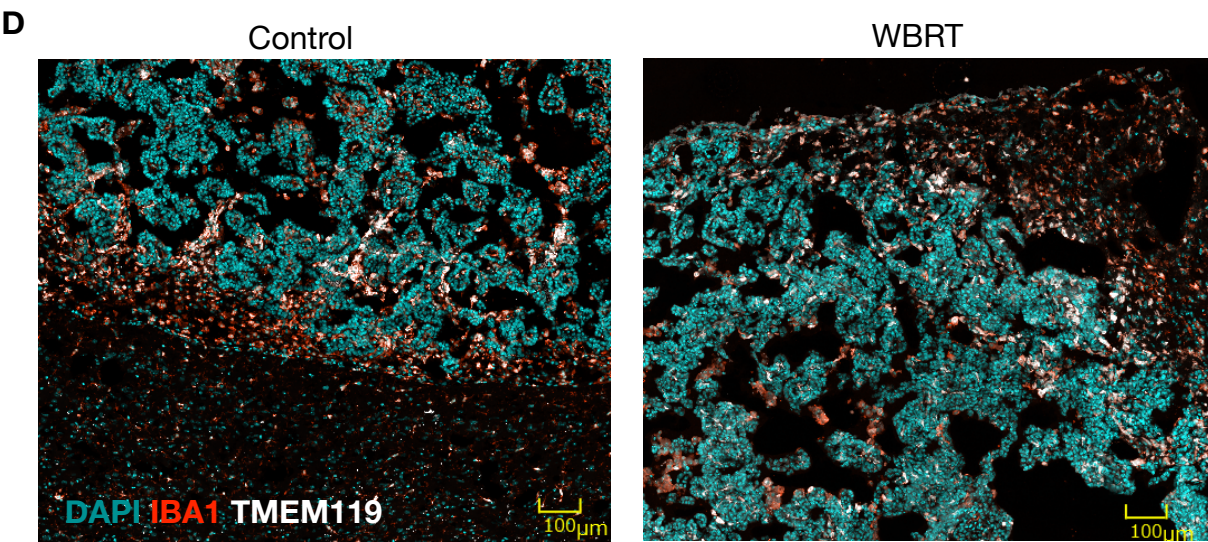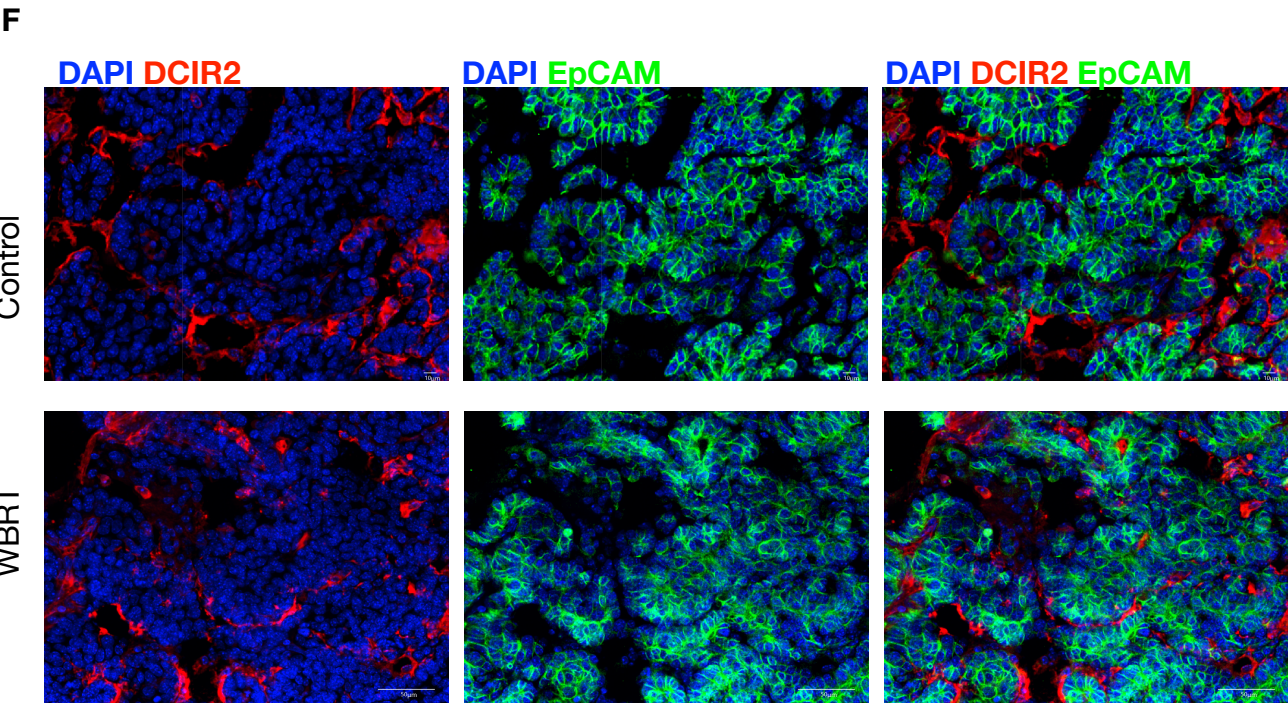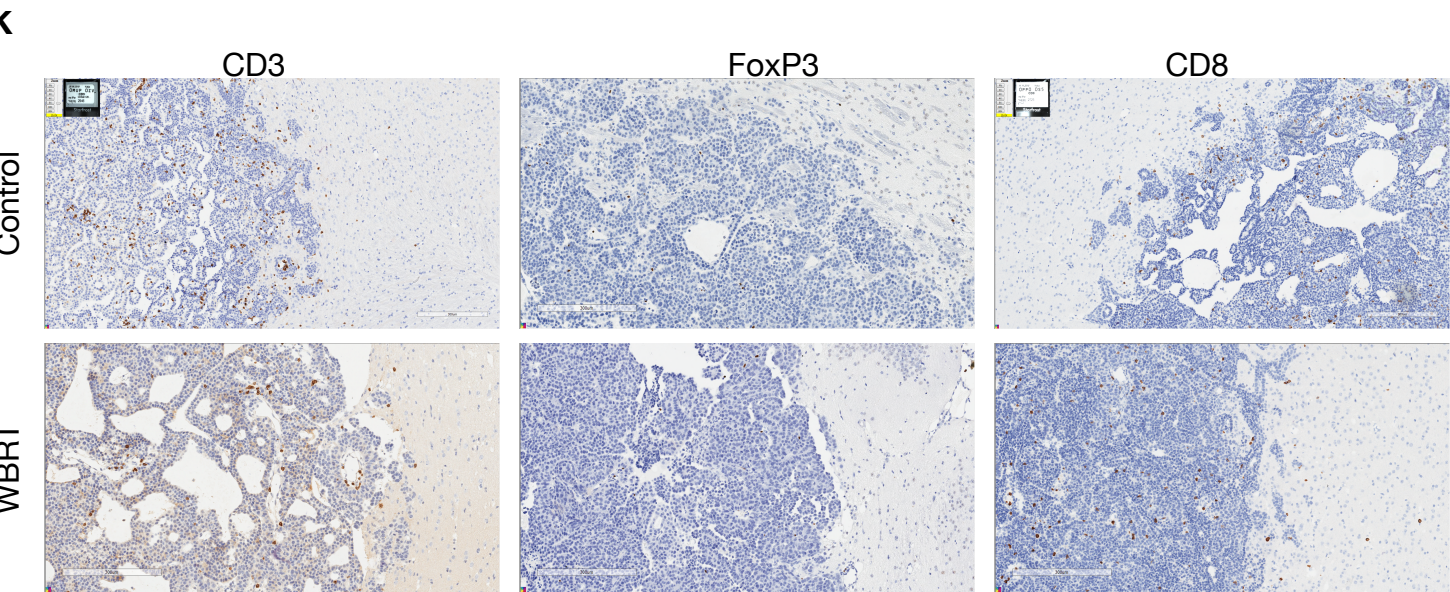

Supplement: Supplementary file 10 — Source Data for Figure 4 [file EMMM-13-e13412-s005.zip › EMM-2020-13412-V2-Figure_4_Source_Data-sd.pdf]
