# Supplementary material for: The immune suppressive microenvironment affects efficacy of radio‐immunotherapy in brain metastasis
Source: EMBO Mol Med. 2021 Mar 23;13(5):e13412. doi: 10.15252/emmm.202013412 (PMC8103101; doi:10.15252/emmm.202013412)

Figure EV2C - uncropped images

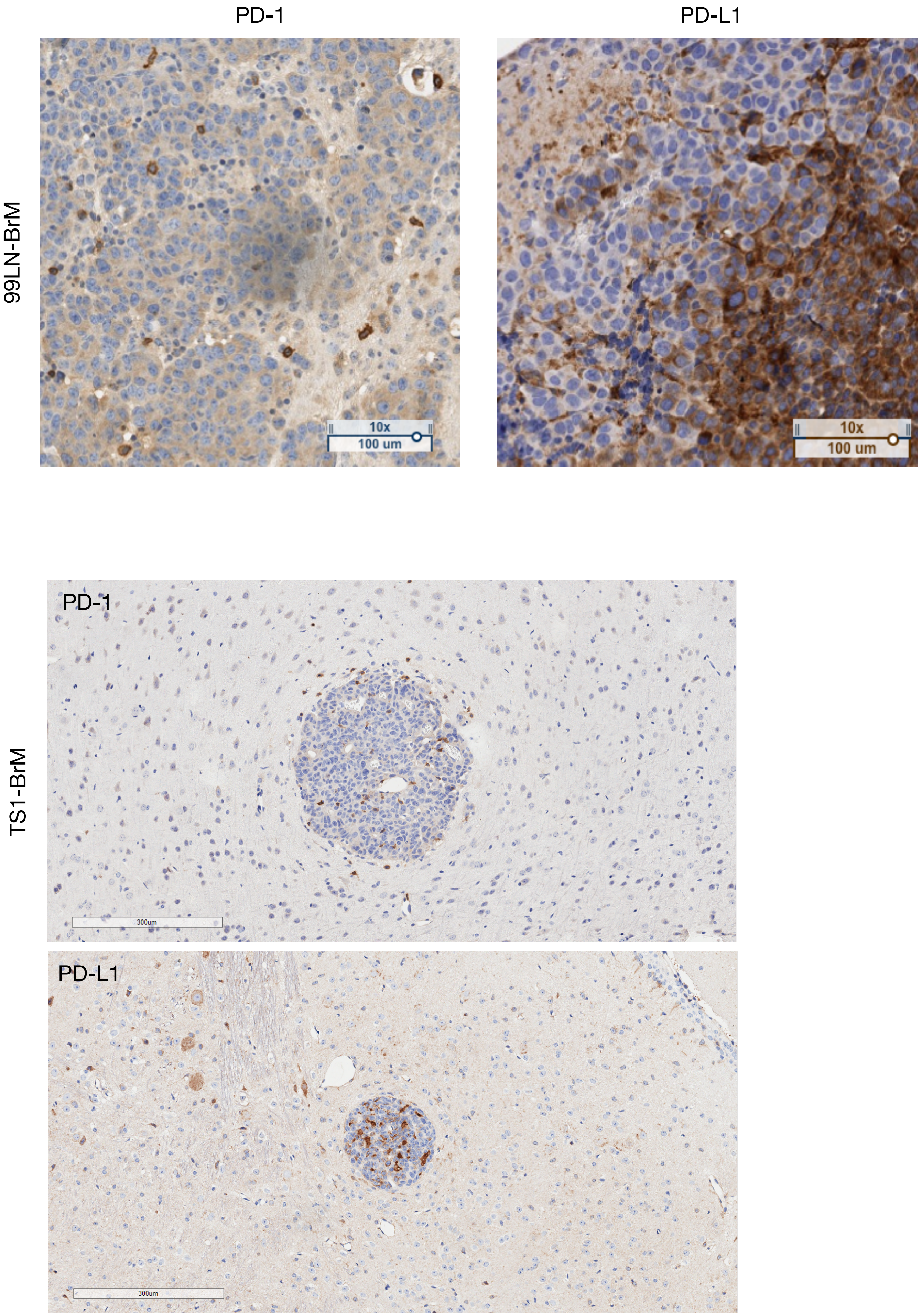

Supplement: Supplementary file 5 — Source Data for Expanded View [file EMMM-13-e13412-s007.zip › EMM-2020-13412-V2-Figure_EV2_Source_Data-sd.zip!/EMM-2020-13412-V2-Figure_EV2_Source_Data-sd.pdf]
